# Antibiotic resistance of stroke associated pneumoni in ICU Kariadi Hospital on 2022 – 2023

**DOI:** 10.1017/ash.2025.162

**Published:** 2025-09-03

**Authors:** Gita Fajar Wardhani, Retnaningsih, Muchlis A.U.Sofro

**Affiliations:** Neurology Resident Diponegoro University/ Kariadi Hospital, Neurology Departement Lecturer Diponegoro University/ Kariadi Hospital and Internal Medicine Department Lecturer Diponegoro University/ Kariadi Hospital

**Keywords:** stroke associated pneumoni, antibiotic resistance, ICU

## Abstract

**Introduction:** Stroke is the second leading cause of death worldwide, accounting for 4.4 million (9%) of the total 50.5 million deaths per year. Infections are among the highest complicating factors of stroke, particularly respiratory tract infections occurring in 23-65% of stroke patients. Stroke complications such as pneumonia and sepsis require ICU care and the use of antibiotics, which poses a risk of antibiotic resistance. The aim of this study is to determine the profile of antibiotic resistance and the factors influencing antibiotic resistance in pneumonia among stroke patients treated in the ICU. **Method:** This study uses a retrospective descriptive method involving 84 stroke patients with pneumonia treated at the ICU of RSUP Dr. Kariadi Semarang from January 2022 to December 2023. **Results:** Among stroke patients with pneumonia, 62 patients (73.8%) developed sepsis, with 64.5% of them experiencing antibiotic resistance. There was no significant difference in antibiotic resistance between stroke patients with pneumonia and those without pneumonia (p = 0.382). There was also no significant difference in antibiotic resistance between pneumonia patients with stroke who developed sepsis and those who did not (p = 0.756). The most commonly found bacteria were *A. baumannii, P. aeruginosa,* and *K. pneumoniae*. The antibiotic most commonly showing resistance was Ampicillin. Patients with diabetes mellitus had a 5.2 times higher risk of experiencing antibiotic resistance. **Conclusion:** Antibiotic resistance can occur in stroke patients with pneumonia and those progressing to sepsis. Diabetes mellitus is a significant risk factor for antibiotic resistance. Antibiotic management programs and infection control are needed to prevent antibiotic resistance in the ICU.

